# Outcome of Elderly Patients with Meningioma after Image-Guided Stereotactic Radiotherapy: A Study of 100 Cases

**DOI:** 10.1155/2015/868401

**Published:** 2015-05-26

**Authors:** David Kaul, Volker Budach, Lukas Graaf, Johannes Gollrad, Harun Badakhshi

**Affiliations:** Department for Radiation Oncology, Charité School of Medicine and University Hospital Berlin, Augustenburger Platz 1, 13353 Berlin, Germany

## Abstract

*Introduction*. Incidence of meningioma increases with age. Surgery has been the mainstay treatment. Elderly patients, however, are at risk of severe morbidity. Therefore, we conducted this study to analyze long-term outcomes of linac-based fractionated stereotactic radiotherapy (FSRT) for older adults (aged ≥65 years) with meningioma and determine prognostic factors. *Materials and Methods*. Between October 1998 and March 2009, 100 patients (≥65, median age, 71 years) were treated with FSRT for meningioma. Two patients were lost to follow-up. Eight patients each had grade I and grade II meningiomas, and five patients had grade III meningiomas. The histology was unknown in 77 cases (grade 0). *Results*. The median follow-up was 37 months, and 3-year, 5-year, and 10-year progression-free survival (PFS) rates were 93.7%, 91.1%, and 82%. Patients with grade 0/I meningioma showed 3- and 5-year PFS rates of 98.4% and 95.6%. Patients with grade II or III meningiomas showed 3-year PFS rates of 36%. 93.8% of patients showed local tumor control. Multivariate analysis did not indicate any significant prognostic factors. *Conclusion*. FSRT may play an important role as a noninvasive and safe method in the clinical management of older patients with meningioma.

## 1. Introduction

Meningioma is the second most common primary brain tumor. It arises from the cap cells of the arachnoid membrane and occurs more frequently in women than men [1].

The incidence of meningioma increases with increasing age. While the incidence rate in 45–54 year olds is 4.9/100,000, this increases to 7.9/100,000 and 12.8/100,000 in 55–64 year olds and those of ≥65 years of age, respectively [2]. A high percentage of meningiomas diagnosed present as small, slow growing, asymptomatic tumors without brain edema; especially in the elderly these cases are often offered conservative clinical observation and radiologic follow-up [3, 4]. However, once the tumors become clinically symptomatic, treatment is needed.

Surgery has traditionally been the mainstay of treatment of symptomatic and fast growing tumors in all age groups. It has obvious advantages in terms of removal of an expansively growing mass. It also allows histological diagnosis, significantly reduces neurological symptoms, and is associated with long-term local tumor control [5, 6].

However, elderly patients may be at risk of severe complications, due to limited physiological capacities and the presence of comorbidities. A recent meta-analysis of the effects of surgery in the older population reported that the overall rates of complications ranged from 2.7% to 29.8%, and the overall incidence of complications was 20% (range, 3–61%) [7]. These findings indicate the need for careful consideration when deciding to perform surgery on older patients. Balancing the potential risks of surgery with the benefits of alternative noninvasive procedures, including image-guided high precision stereotactic radiotherapy (SRT), is important for multidisciplinary decision making with regard to the choice of treatment modality. In addition, only few studies have evaluated the efficacy and benefits of radiotherapy for this patient population.

In this monocentric study, we critically analyzed the feasibility of treatment and clinical outcomes, including tumor control and survival, in older patients (≥65 years) with meningioma treated with linac-based fractionated SRT (FSRT) to evaluate the advantages and limitations of SRT for this particular patient group.

## 2. Material and Methods

### 2.1. Treatment Decisions, Patient Selection, and Dose Regimens

Our local ethics committee approved this study. The research complied with the Helsinki Declaration. We performed a retrospective analysis of 100 elderly patients (≥65) who underwent SRT of an intracranial meningioma between 10/1998 and 03/2009. Two patients were lost to follow-up. Follow-up data were analyzed until March 2010.

All patients underwent computed tomography and magnetic resonance imaging including a diffusion-weighted series of the head. All images were evaluated by a neuroradiologist. In our institution an interdisciplinary team encompassing radiation oncologists, neurosurgeons, pathologists, and radiologists makes treatment decisions. Adjuvant SRT is offered to all resected grade II and III meningioma patients; symptomatic grade I meningiomas are treated with adjuvant RT only after incomplete resection or when recurrence occurs after total resection. Clinically symptomatic and fast growing tumors that are considered inoperable either by the anesthesiology department due to comorbidities or by the neurosurgery department due to difficult localization are treated using primary FSRT. Tumors are classified according to the Novel “CLASS” Algorithmic Scale for Patient Selection in Meningioma Surgery: low risk, medium risk, high risk, and optical nerve sheath (ONSM) [8]. High-risk patients usually receive FSRT rather than surgical treatment.

1.6–2 Gy were considered normofractionated (nFSRT), 2.8–5 Gy were considered hypofractionated (hFSRT), and high single doses delivered in less than 5 sessions were considered stereotactic radiosurgery (SRS). Tumors in close proximity to critical structures were assigned to nFSRT, while large tumors (>2 cm) distant to critical structures underwent hFSRT and small tumors (<2 cm) were treated by SRS.

### 2.2. Stratification and Variables

Patients were stratified according to grading, localization (skull base, falx/parasagittal, and convexity), predicted perioperative risk/operability, tumor size, and sequence of therapy. Two groups were defined: group 1 encompasses all grade I meningiomas, as well as all meningiomas with no histology available (grade 0). Group 2 encompasses all grade II and III meningiomas.

The tumor location was divided into 3 groups: skull base, falx/parasagittal, and convexity.

Follow-up examinations, including MRI as well as clinical and neurologic examinations, were performed at 6 weeks, 3 months, 9 months, and 15 months after treatment and then annually.

We distinguished between primary radiation treatment and postoperative radiotherapy. Acute toxicity in the first 90 days after FSRT was graded using a modified version of the Common Terminology Criteria of Adverse Events (CTCAE v4.0).

### 2.3. Technical Set-Up

From 1995–2003 meningioma patients underwent “sharp” fixation using a stereotactic head ring and an oral bite plate. A 6 MV Linac (Varian USA) with an add-on micro-multileaf collimator (mMLC) (BrainLab Co, Germany) was used. Coordinates for SRS were set by a laser-based stereotactic localizer. This set-up allowed delivering shaped beams. In 2004 we started using Novalis (BrainLab) with beam shaping capability using build-in MLC and image guidance. Novalis ExacTrac image-guided frameless system enabled us to image the patient at any couch position using a frameless positioning array. MRI/CT-fusion planning was performed. The three-dimensional treatment planning system Brainscan (Brain Lab AG, Germany) was used, which was later replaced by iplanRT. The gross tumor volume (GTV) was defined as the area of contrast enhancement on T1-weighted MRI images; the planning target volume (PTV) included a 2 mm isotropic safety margin. The dose was prescribed to a reference point, which was the isocenter (or the center of GTV), though 100% was not the maximum dose but the dose at the aforementioned reference point. Patients received the prescribed dose to the 95% isodose at the tumor margin. Organs at risk (OAR), such as optic nerves, the chiasm, lenses, and the brainstem, were delineated. Dose constraints were according to the data published by Emami et al. 1991 [9]. The TD 5/5 to be respected was as follows: for optic nerves 50 Gy, for chiasm 50 Gy, for lenses 10 Gy, and for the brainstem 50 Gy, respectively.

### 2.4. Statistics

All statistical analyses were performed using IBM SPSS Statistics 19 (New York, USA).

## 3. Results

### 3.1. Patient Characteristics

An initial review of medical records revealed 100 cases of older patients (≥65) who received FSRT for meningioma. Two patients were lost to follow-up in the early posttherapeutic period. The remaining 98 patients included 62 women and 36 men. Histology was unknown for 77.6% of patients, 8.2% of patients presented with World Health Organization (WHO) grade I lesions, 8.2% presented with grade II lesions, and 5.1% were diagnosed as grade III meningioma. The majority of the lesions were located in the skull base (79.6%), 8.2% were located in the falx or parasagittal region, and 12.2% were meningiomas of the convexity. The median follow-up period following treatment was 37 months. Patient characteristics are summarized in Table 1.

### 3.2. Progression-Free Survival and Univariate and Multivariate Analysis

**Figure 1:**
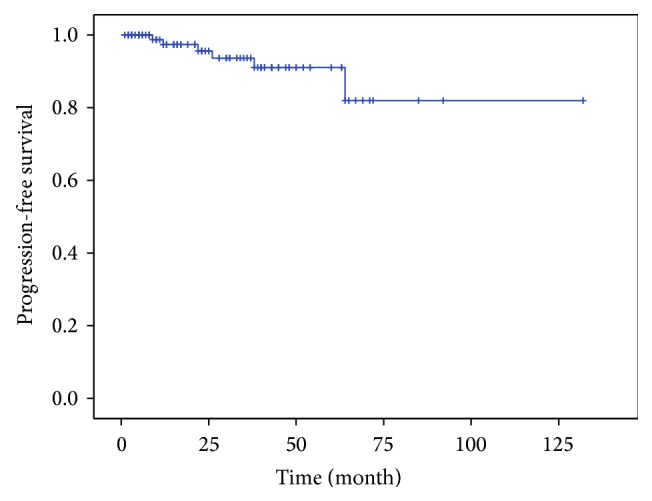
PFS rates of the entire cohort. PFS rates were 93.7% after 3 years, 91.1% after 5 years, and 82% after 10 years.

**Figure 2:**
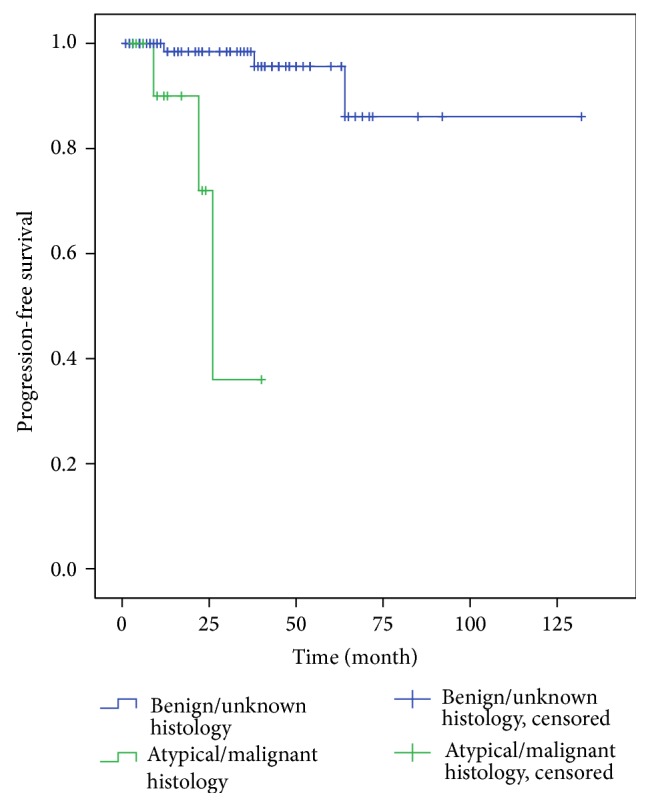
PFS rates of group 1 and group 2. Patients with grade I meningioma or unknown histology showed PFS rates of 98.4% and 95.6% at 3 and 5 years, respectively; patients with grade II or III meningioma showed PFS rates of 36% after 3 years (*P* < 0.0001).

The results of univariate analysis (UVA) and multivariate analysis (MVA) of predictive factors are shown in Table 2. In the entire cohort, 3-year, 5-year, and 10-year progression-free survival (PFS) rates were 93.7%, 91.1%, and 82%, respectively (Figure 1). Patients with grade I meningioma or unknown histology (grade 0) had 3-year and 5-year PFS rates of 98.4% and 95.6%, respectively, while patients with grade II or III meningioma showed a 3-year PFS rate of 36% (Figure 2). The difference in PFS rates between the grade 0/I group and the grade II/III group was statistically significant using the Log-Rank Test (*P* < 0.0001).

**Figure 3:**
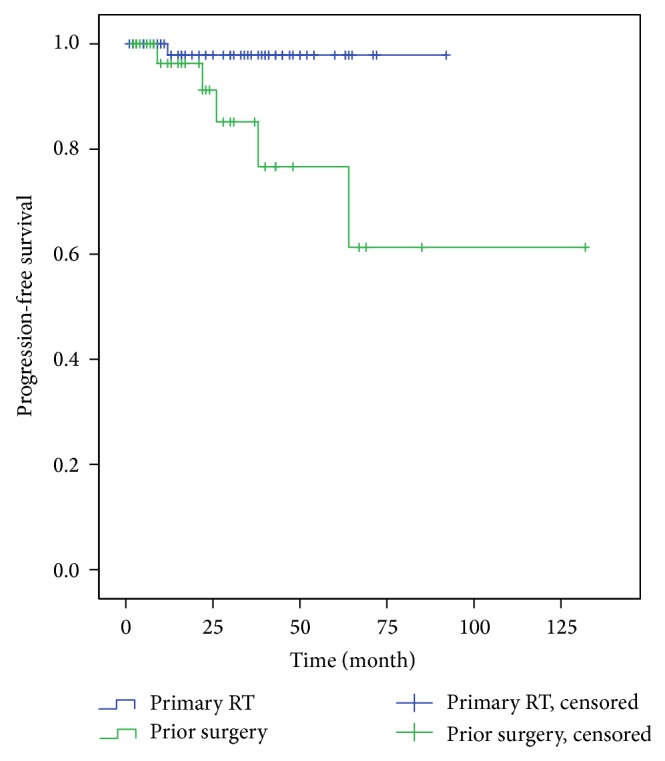
PFS rates for patients with primary and postoperative FSRT. There was a significant difference in PFS rates for patients treated with primary and adjuvant FSRT (*P* < 0.01 Log-Rank Test).

The 3-year and 5-year PFS rates for patients who had not undergone prior surgery were both 97.9%. The difference in PFS rates between the patients who had undergone prior surgery and the patients who had not received surgical treatment was significant using the Log-Rank Test (*P* < 0.01, Figure 3).

Patients with a target volume of <6.4 cm^3^ did not show significantly improved PFS rates compared to those with target volumes >6.4 cm^3^. PFS rates were independent of age (>71 versus ≤71 years), sex (male versus female), location of tumor, and fractionation scheme. None of the factors analyzed showed significant predictive value on multivariate analysis.

### 3.3. Radiologic Response

Radiologic response rates are shown in Table 3. Ninety-two patients (93.8%) showed local tumor control, 21.4% of which showed tumor regression. Only 6.1% of patients showed local tumor progression.

### 3.4. Acute Toxicity

Acute toxicity data were available for 88 patients (89.8%), and of these patients, 53 (54.1%) showed acute toxicity. The most common acute grade I symptoms for the entire cohort were headache, fatigue, and local alopecia. The most common acute grade II symptoms were vertigo, headache, and local alopecia.

### 3.5. Chronic Toxicity

Late toxicity data were available for 98 patients (100%), and of these patients, 16 (16.3%) showed late toxicity. The most common grade I symptoms were fatigue, local alopecia, and headache. No grade II or III symptoms were found.

## 4. Discussion

Increased incidence of intracranial meningioma correlates with increasing age [2, 10–14]. It is widely accepted that a demographic shift toward an ageing population is occurring worldwide, and this will lead to an expected increase in the incidence of meningioma. While some data on surgical treatment of meningioma are available [7], only few reports focusing on the safety and efficacy of radiotherapy in older adults have been published. Therefore, this study aimed to explore the potential utility of a noninvasive therapeutic procedure, high-precision image-guided FSRT, with regard to feasibility, safety, and clinical outcomes in older patients with meningioma [15–18].

This study shows that FSRT is feasible on the procedural level and is safe with regard to toxicity. Furthermore, noninvasive FSRT was effective in terms of tumor control and survival for this ever-expanding patient group. Our results indicate that older patients (aged ≥65) may benefit from FSRT for the treatment of meningiomas.

In the entire cohort, 3-year, 5-year, and 10-year progression-free survival (PFS) rates were 93.7%, 91.1%, and 82%, respectively. This is in accordance with a recent study carried out by Fokas et al. of 121 cases of meningioma with a similar follow-up time (40 months) reporting local control rates of 98.3% at 1 and 3 years and 94.7% at 5 years [15].

We carried out UVA to examine the prognostic relevance of clinical factors (Table 2) and found that tumor localization, prior surgery, and grade had an association with prognosis (*P* < 0.0001, *P* < 0.01, and *P* < 0.0001, resp.) in UVA. However, in agreement with the findings of Fokas et al., (MVA, Table 2) no significant prognostic associations with age, sex, grade, tumor localization, target volume, radiotherapy regimen, or prior surgery were found in MVA.

With regard to toxicity outcomes, our results indicate the safety of this treatment modality for the older population, who is at risk of higher treatment-related complications due to lower performance indices and comorbidities. Reports of several surgical series in older adults have found that the incidence of associated morbidity ranges from 9% to 54% in this population [19–22]. The largest surgical series that examined outcome in 258 older patients with meningioma indicated morbidity rates of 29.8% [20]. Schul et al. published outcome data for surgically treated patients and reported a 21% rate of surgery-related morbidity [21]. Similar numbers (17.8%) were reported by Boviatsis et al. [23]. This study, in agreement with other studies of older patients, found that FSRT, in contrast to surgical treatment, is a safe and effective treatment modality for meningioma in the older population.

Our study had some limitations. Firstly the retrospective nature of the analysis is prone to bias. Secondly, the median follow-up was only 37 months and it is known that late recurrences do occur in meningioma patients even after 5 years. The number of patients with more than five years of follow-up was *n* = 13. Thirdly the treatment heterogeneity must be mentioned here, as different fractionation regimens were used.

In conclusion, this is one of the first large studies to evaluate feasibility, safety, and the efficacy of FSRT in older patients with meningioma. We demonstrated the safety and efficacy of SRT in this particular patient group. The demographic shift towards and ageing population requires innovative disease management. Radiotherapy may play an important role as a noninvasive, safe, and relatively cost-effective method in the clinical management of older patients with meningioma.

## Figures and Tables

**Table 1:** Patient characteristics.

	Overall collective	
	(*n* = 98)	
	Median	Min/max
Age with beginning of RT	71	65/87
Tumor volume	6.4	0.26/86.39

	*n*	%

Gender		
m	36	36.7
f	62	63.3
Location		
Skull base	78	79.6
Falx/parasagittal	8	8.2
Convexity	12	12.2
WHO grading		
n/a	77	77.6
WHO 1	8	8.2
WHO 2	8	8.2
WHO 3	5	5.1
Prior surgery		
Primary RT	61	62.2
Adjuvant RT	37	37.8
Peritumoral edema		
Yes	4	4.1
Multiple meningiomas		
Yes	26	26.5
Fractionation scheme		
FSRT	50	51
hFSRT	38	38.8
SRS	10	10.2

	Median	Min/max

FSRT total dose (Gy)	56.5	28.8/72
hFSRT total dose (Gy)	36.3	30/42
SRS total dose (Gy)	17.6	13.5/21.4

Follow-up time in months	37	1/132

**Table 2:** Univariate and multivariate analysis of PFS rates. In univariate analysis localization, prior surgery and grading showed a significant effect on prognosis (*P* < 0.0001, *P* < 0.01, and *P* < 0.0001); however multivariate analysis could not confirm these findings.

	Univariate	Multivariate			
	*P* value	HR	95% CI		*P* value
			Lower	Upper	
Age (≤71 versus >71 years)	0.24	2.87	0.32	25.43	0.35
Sex (m versus f)	0.36	3.67	0.37	35.90	0.26
Grading (unknown histology and grade I versus grade II/III)	**0.0001**	9.646	0.383	242.687	0.168
Localization (skull base, falx/parasagittal, and convexity)	**0.0001**	1.64	0.25	10.59	0.603
Target volume (≤6.4 versus >6.4 ccm)	0.54	3.41	0.38	30.72	0.27
RT regimen (FSRT versus hSRT versus SRS)	0.32	1.40	0.30	6.56	0.67
Prior surgery	**0.01**	4.49	0.28	71.64	0.29

**Table 3:** Tumor response rates.

	Frequency	%
Progression	6	6.1
Stable disease	71	72.4
Regression	21	21.4

## References

[1] Kaul D., Budach V., Wurm R. (2014). Linac-based stereotactic radiotherapy and radiosurgery in patients with meningioma. *Radiation Oncology*.

[2] Claus E. B., Bondy M. L., Schildkraut J. M., Wiemels J. L., Wrensch M., Black P. M. (2005). Epidemiology of intracranial meningioma. *Neurosurgery*.

[3] Stessin A. M., Schwartz A., Judanin G. (2012). Does adjuvant external-beam radiotherapy improve outcomes for nonbenign meningiomas? A Surveillance, Epidemiology, and End Results (SEER)-based analysis. *Journal of Neurosurgery*.

[4] Komotar R. J., Bryan Lorgulescu J., Raper D. M. S. (2012). The role of radiotherapy following gross-total resection of atypical meningiomas. *Journal of Neurosurgery*.

[5] Pechlivanis I., Wawrzyniak S., Engelhardt M., Schmieder K. (2011). Evidence level in the treatment of meningioma with focus on the comparison between surgery versus radiotherapy: a review. *Journal of Neurosurgical Sciences*.

[6] Cahill K. S., Claus E. B. (2011). Treatment and survival of patients with nonmalignant intracranial meningioma: results from the surveillance, epidemiology, and end results program of the National Cancer Institute. Clinical article. *Journal of Neurosurgery*.

[7] Poon M. T.-C., Fung L. H.-K., Pu J. K.-S., Leung G. K.-K. (2014). Outcome of elderly patients undergoing intracranial meningioma resection—a systematic review and meta-analysis. *British Journal of Neurosurgery*.

[8] Lee J., Sade B., Lee J. (2009). The novel ‘CLASS’ algorithmic scale for patient selection in meningioma surgery. *Meningiomas*.

[9] Emami B., Lyman J., Brown A. (1991). Tolerance of normal tissue to therapeutic irradiation. *International Journal of Radiation Oncology, Biology, Physics*.

[10] Bateman B. T., Pile-Spellman J., Gutin P. H., Berman M. F. (2005). Meningioma resection in the elderly: Nationwide inpatient sample, 1998–2002. *Neurosurgery*.

[11] Dolecek T. A., Propp J. M., Stroup N. E., Kruchko C. (2012). CBTRUS statistical report: primary brain and central nervous system tumors diagnosed in the United States in 2005–2009. *Neuro-Oncology*.

[12] Kondziolka D., Levy E. I., Niranjan A., Flickinger J. C., Lunsford L. D. (1999). Long-term outcomes after meningioma radiosurgery: physician and patient perspectives. *Journal of Neurosurgery*.

[13] Stafford S. L., Pollock B. E., Foote R. L. (2001). Meningioma radiosurgery: tumor control, outcomes, and complications among 190 consecutive patients. *Neurosurgery*.

[14] Taylor B. W., Marcus R. B., Friedman W. A., Ballinger W. E., Million R. R. (1988). The meningioma controversy: postoperative radiation therapy. *International Journal of Radiation Oncology, Biology, Physics*.

[15] Fokas E., Henzel M., Surber G., Hamm K., Engenhart-Cabillic R. (2014). Stereotactic radiotherapy of benign meningioma in the elderly: clinical outcome and toxicity in 121 patients. *Radiotherapy and Oncology*.

[16] Igaki H., Maruyama K., Koga T. (2009). Stereotactic radiosurgery for skull base meningioma. *Neurologia Medico-Chirurgica*.

[17] Iwai Y., Yamanaka K., Ikeda H. (2008). Gamma knife radiosurgery for skull base meningioma: long-term results of low-dose treatment. *Journal of Neurosurgery*.

[18] Kalogeridi M.-A., Georgolopoulou P., Kouloulias V., Kouvaris J., Pissakas G. (2010). Long-term follow-up confirms the efficacy of Linac radiosurgery for acoustic neuroma and meningioma patients. A single institution's experience. *Journal of B.U.ON.*.

[19] Tucha O., Smely C., Lange K. W. (2001). Effects of surgery on cognitive functioning of elderly patients with intracranial meningioma. *British Journal of Neurosurgery*.

[20] Patil C. G., Veeravagu A., Lad S. P., Boaky M. (2010). Craniotomy for resection of meningioma in the elderly: a multicentre, prospective analysis from the national surgical quality improvement program. *Journal of Neurology, Neurosurgery and Psychiatry*.

[21] Schul D. B., Wolf S., Krammer M. J., Landscheidt J. F., Tomasino A., Lumenta C. B. (2012). Meningioma surgery in the elderly: outcome and validation of 2 proposed grading score systems. *Neurosurgery*.

[22] Locatelli M., Bertani G., Carrabba G. (2013). The trans-sphenoidal resection of pituitary adenomas in elderly patients and surgical risk. *Pituitary*.

[23] Boviatsis E. J., Bouras T. I., Kouyialis A. T., Themistocleous M. S., Sakas D. E. (2007). Impact of age on complications and outcome in meningioma surgery. *Surgical Neurology*.

